# ^1^H-NMR Metabolite Fingerprinting Analysis Reveals a Disease Biomarker and a Field Treatment Response in *Xylella fastidiosa* subsp. *pauca*-Infected Olive Trees

**DOI:** 10.3390/plants8050115

**Published:** 2019-04-29

**Authors:** Chiara Roberta Girelli, Federica Angilè, Laura Del Coco, Danilo Migoni, Luigi Zampella, Simone Marcelletti, Nicola Cristella, Paolo Marangi, Marco Scortichini, Francesco Paolo Fanizzi

**Affiliations:** 1 Department of Biological and Environmental Sciences and Technologies, University of Salento, Prov.le Lecce-Monteroni, I-73100 Lecce, Italy; chiara.girelli@unisalento.it (C.R.G.); federica.angile@unisalento.it (F.A.); laura.delcoco@unisalento.it (L.D.C.); danilo.migoni@unisalento.it (D.M.); 2 Council for Agricultural research and Economics-Research Centre for Olive, Fruit Trees and Citrus, Via Torrino, 3, I-81100 Caserta, Italy; luigizampella@live.it (L.Z.); simone._82@hotmail.it (S.M.); marco.scortichini@crea.gov.it (M.S.); 3 Studio Agro-Ambientale ed Ingegneria Terranostra srls, Via XXIV Maggio, 10, I-74020 Lizzano, Italy; nicolacristella@gmail.com (N.C.); marangi1974@libero.it (P.M.)

**Keywords:** metabolomics, multivariate statistical analysis, quinic acid, polyphenols, malic acid, GABA

## Abstract

*Xylella fastidiosa* subsp. *pauca* is a xylem-limited bacterial phytopathogen currently found associated on many hectares with the “olive quick decline syndrome” in the Apulia region (Southern Italy), and the cultivars Ogliarola salentina and Cellina di Nardò result in being particularly sensitive to the disease. In order to find compounds showing the capability of reducing the population cell density of the pathogen within the leaves, we tested, in some olive orchards naturally-infected by the bacterium, a zinc-copper-citric acid biocomplex, namely Dentamet®, by spraying it to the crown, once per month, during spring and summer. The occurrence of the pathogen in the four olive orchards chosen for the trial was molecularly assessed. A ^1^H NMR metabolomic approach, in conjunction with a multivariate statistical analysis, was applied to investigate the metabolic pattern of both infected and treated adult olive cultivars, Ogliarola salentina and Cellina di Nardò trees, in two sampling periods, performed during the first year of the trial. For both cultivars and sampling periods, the orthogonal partial least squares discriminant analysis (OPLS-DA) gave good models of separation according to the treatment application. In both cultivars, some metabolites such as quinic acid, the aldehydic form of oleoeuropein, ligstroside and phenolic compounds, were consistently found as discriminative for the untreated olive trees in comparison with the Dentamet®-treated trees. Quinic acid, a precursor of lignin, was confirmed as a disease biomarker for the olive trees infected by *X. fastidiosa* subsp. *pauca.* When treated with Dentamet®, the two cultivars showed a distinct response. A consistent increase in malic acid was observed for the Ogliarola salentina trees, whereas in the Cellina di Nardò trees the treatments attenuate the metabolic response to the infection. To note that in Cellina di Nardò trees at the first sampling, an increase in γ-aminobutyric acid (GABA) was observed. This study highlights how the infection incited by *X. fastidiosa* subsp. *pauca* strongly modifies the overall metabolism of olive trees, and how a zinc-copper-citric acid biocomplex can induce an early re-programming of the metabolic pathways in the infected trees.

## 1. Introduction

*Xylella fastidiosa* is a xylem-limited phytopathogen spread to cultivated crops and wild plants through the feeding activity of some insect vectors, and as a major consequence of infection, it causes the blockage of the xylem vessels by forming biofilms [1]. Due to its dangerousness, it is currently included into the A2 list of the quarantine bacteria for the European Plant Protection Organization (EPPO). Within the species, there are recognized and described three subspecies, namely *fastidiosa*, *multiplex* and *pauca*, each of them showing typical molecular and pathogenic features [2,3,4]. In the Apulia region (southern Italy), the subspecies *pauca* was found associated with the “olive quick decline syndrome” (OQDS), a severe disease currently affecting many olive orchards. Main symptoms include leaf, twig and branch dieback, often followed by a generalized collapse of the plant [5]. In the infection disease process incited by *X. fastidiosa* to many crops, both plant micronutrients and secondary metabolites, such as the phenolic compounds, would seem to play relevant roles. Among micronutrients, the concentration of ions, such as zinc and copper, is linked to the multiplication of the pathogen, so that copper and zinc concentration higher than 200 µM and 0.25 mM, respectively, inhibits *X. fastidiosa* biofilm formation [6]. On the other hand, it is known that zinc detoxification *in planta* is required for the full virulence of the pathogen [7]. In addition, in *Vaccinium corimbosum* hybrid plants artificially inoculated with strains of *X. fastidiosa* subsp. *Multiplex*, and in olive trees naturally infected by *X. fastidiosa* subsp. *pauca*, a relevant decrease of copper content in the leaf was observed [8,9]. Concerning phenolic compounds, the xylem sap of grapevine plants infected by *X. fastidiosa* subsp. *fastidiosa* showed a relevant increase of catechins, digalloyiquinic acid and astringin, whereas high contents of procyanidins, stilbenoids and catechins were found in xylem tissue [10]. However, it should be said that phenology would seem to influence phenolic profile more than the infection status [11]. In addition, grapevine rootstocks can affect the xylem sap phenolic content [12]. In olive trees naturally infected by *X. fastidiosa* subsp. *pauca*, regardless of the cultivar tested, the content of quinic acid in the leaves is significantly higher than the content of healthy plants [13]. Moreover, lignin accumulation in the xylem tissue would seem to play a relevant role in the tolerance of the olive cultivar “Leccino” to the pathogen [14].

Among the analytical techniques available for deciphering molecules involved in the plant-microbe interaction, metabolomics such as mass spectrometry (MS) and nuclear magnetic resonance (NMR) analyses, could offer several advantages for obtaining an overview of all detectable small metabolites, and for obtaining robust, high-reproducible, spectral data, with repletion of experiments also in different years [15]. NMR in plants metabolomics has a wide range of applications, such as in the investigations of environmental factors: Growth conditions, exposure to pesticides, agronomic practices [16]. The combination of NMR and chemometrics can help to characterize metabolic patterns in plants under stress conditions, such as pathogen infections, and could be useful to understand plant physiology, which is pivotal for the future applications in disease control [17].

Previously, in an attempt to find out possible compounds showing the capacity to reduce the concentration of *X. fastidiosa* subsp. *pauca* within olive leaves, we tested a zinc-copper-citric acid biocomplex in an olive orchard planted with the sensitive cultivars Ogliarola salentina and Cellina di Nardò, naturally infected by the bacterium [18]. This patented foliar fertilizer is composed of zinc (4% *w*/*w*) and copper (2% *w*/*w*), it has a specific weight of 1.280 g/L, and it is obtained through an electrolytic process by complexing the ions with hydracids of citric acid obtained through the fermentation of soil fungi. It is sprayed into the olive trees’ crowns once per month from March-April to September-October, at a dose of 3.9 L/ha, and can also be used in organic agriculture. In parallel, through ^1^H NMR and multivariate statistical analysis, we also preliminarily assessed the metabolomic profiles of extracts obtained from the leaves of the two cultivars, and we found an opposite trend concerning the sugars and polyphenols content [19]. In this study, an ^1^H NMR-based metabolomic approach was used to provide a snapshot of the *X. fastidiosa*-infected Ogliarola salentina and Cellina di Nardò in comparison with trees which have received some treatment with the zinc-copper-citric acid biocomplex. Potential biomarkers of the disease were also identified by comparing the metabolomic profiles of leaf samples taken from untreated and treated trees of the two olive cultivars.

## 2. Results

### 2.1. Detection of Xylella Fastidiosa and Field Symptoms

The occurrence of *X. fastidiosa* was molecularly ascertained in each olive orchard, thus confirming previous analyses indicating the presence of the pathogen both in Veglie and Martina Franca orchards as revealed by the Phytosanitary Service of Apulia Region. In each location, both untreated Ogliarola salentina and Cellina di Nardò trees showed from April to September 2017 a mean severity of symptoms (i.e., leaf and twig dieback) higher than those observed for the Dentamet®-treated trees (Table 1).

### 2.2. Visual Inspection of ^1^H NMR Spectra

**Figure 1:**
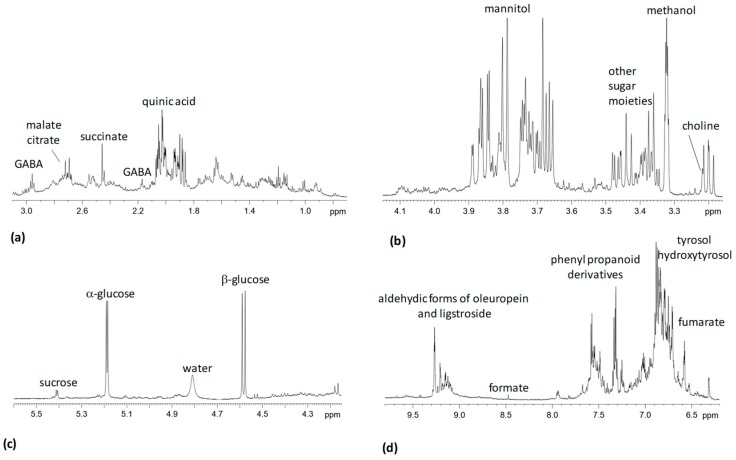
600 MHz typical ^1^H NMR spectrum of olive leaves aqueous extract sample. Expanded area in the range (**a**) 0.5–3.10 ppm; (**b**) 3.15–4.15 ppm; (**c**) 4.2–5.7 ppm and (**d**) 6–10 ppm. Assignment of the main peaks are indicated.

Visual inspection of the 600 MHz ^1^H NMR spectra revealed a complex pattern of signals due to sugars, phenolic compounds such as quinic acid, hydroxytyrosol, tyrosol, phenylpropanoids derivatives and aldehydic secoiridoids (oleuropein and ligstroside) (Figure 1). 

Signals in the region between 3 and 5 ppm correspond mainly to sugars. In particular, mannitol signals in the region between 3.6 and 3.9 ppm could be observed. Moreover, anomeric proton resonances at 5.20 ppm and 4.60 ppm of monosaccharides as α and β-glucose respectively, were identified. Characteristic anomeric proton doublet at 5.41 ppm, assigned to disaccharide sucrose was also observed. *Xylella fastidiosa* use cellulose-degrading enzymes that alter plant cell wall components to release sucrose [20,21]. These enzymes are also required for degradation of the xylem pit membrane and nutrient release. This helps successful colonization of the xylem vessels [21,22]. Mannitol, a natural polyalcohol with six carbon atoms is, together with glucose the most abundant soluble sugar in olive leaves [23]. In addition, to their role as osmoprotectants, sugar alcohols contribute to the protection against salt and photoxidative stress. Moreover, some sugar alcohols, such as mannitol, play a role in plant pathogen interactions [24]. The singlet at 3.22 ppm assigned to choline, an important osmolyte [25], superimposed to β-glucose (3.20 ppm) triplet was identified by correlation *via* HSQC to carbon atom at 56 ppm. In the aliphatic region the secondary metabolite quinic acid was identified at 1.89, 2.02, 4.01, 3.55 ppm. Among amino acids, signals at 3.0 and 2.28 ppm were assigned to γ-aminobutyrate (GABA), a non-protein amino acid widely diffused throughout the biological world. Organic acids as succinate (2.42 ppm), malate (2.66-2.36 ppm), citrate (2.70–2.53 ppm), formate (8.45 ppm), were also identified. Intense NMR signals assigned to phenyl alcohol moieties (tyrosol and hydroxytyrosol) of oleuropein, principal compound in olive leaf extracts [26,27] and ligstroside and their aglycone were observed in the range at δ = 6.55 − 7.10 ppm. NMR signals in the range of δ = 9.0 − 9.5 ppm were attributed to aldehydic and dialdehydic forms of oleuropein and ligstroside [28].

### 2.3. Unsupervised and Discriminant Analyses on Ogliarola Salentina and Cellina di Nardò Trees Naturally-Infected by Xylella fastidiosa

**Figure 2:**
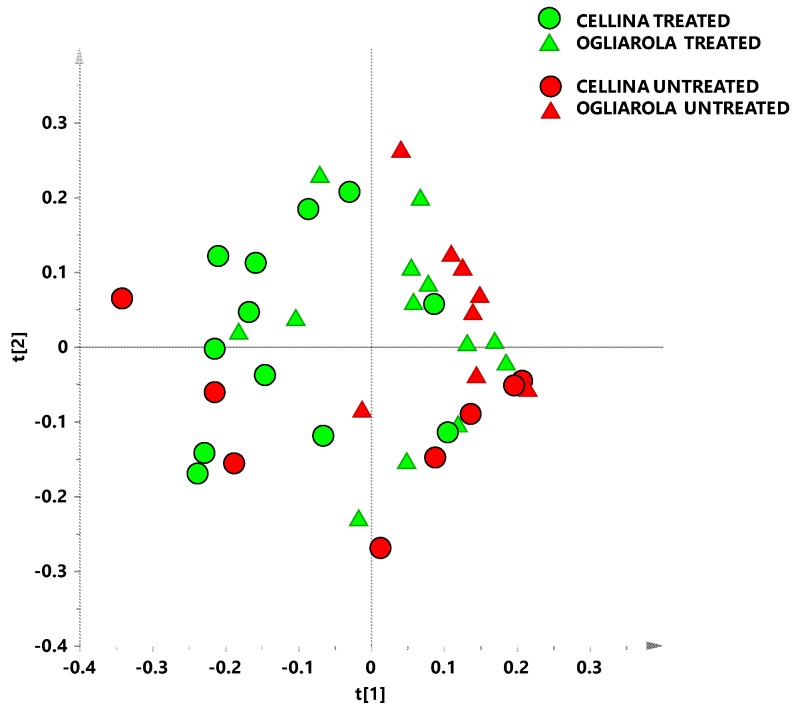
PCA t[1]/t[2] scores plot for Cellina di Nardò untreated (red circles) and treated with Dentamet® (green circles) leaf samples and Ogliarola salentina untreated (red triangles) and treated with Dentamet® leaf samples (green triangles) ((t[1] and t[2] explain 30.2% and 21.2% of the total variance, respectively).).

Individuation problems of molecules responsible for key differences, due to the plethora of signals in a high resolution ^1^H NMR spectrum, can be overcame by using chemometric techniques [29]. This approach allows to obtain a metabolomic fingerprinting of the studied organism and to detect any shift or anomaly in its metabolism [30]. Thus, multivariate statistical analyses were applied to investigate the metabolic profiles of aqueous extracts samples taken from infected Ogliarola salentina and Cellina di Nardò olive trees treated or not with Dentamet®. A preliminary unsupervised analysis was performed on the whole data set in order to observe a general trend of data clustering. The model resulting from the explorative PCA analysis was built with five components (t[1], t[2], t[3], t[4] and t[5] representing 30.2%, 21.2%, 15.4%,11.4% and 5.61% of the total variance, respectively)which gave R^2^X = 0.84 and Q^2^ = 0.691. The scores plot reported in Figure 2 was defined by the first two components t[1] and t[2] that account for 30% and 21% of the explained variance respectively. Visual inspection of the score plot, in which samples from Cellina di Nardò and Ogliarola salentina Dentamet® treated and untreated samples can be observed, revealed a discrimination trend according to the original cultivar. In particular, Ogliarola-treated and untreated samples placed at positive values of t1 component and, most of the Ogliarola treated and untreated samples placed at negative values of t2. Nevertheless, a general dispersion along t2 component, attributable to the sampling period, could be observed.

**Figure 3:**
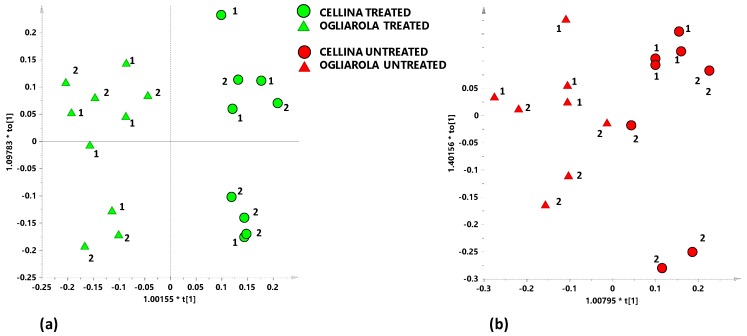
(**a**) OPLS-DA score plot for Ogliarola salentina (green triangles) and Cellina di Nardò (green circles) treated with Dentamet® leaf extracts samples (1+1+0; R^2^X = 0.516; R^2^Y = 0.916; Q^2^ = 0.838); (**b**) OPLS-DA score plot for Ogliarola salentina (red triangles) and Cellina di Nardò (red circles) untreated leaf extracts samples (1+1+0; R^2^X = 0.576; R^2^Y = 0.811; Q^2^ = 0.509).

Discrimination between the two cultivars was then studied by considering separately untreated and Dentamet®-treated samples. Supervised analysis performed on the bucket reduced spectra of Dentamet®-treated and untreated Cellina di Nardò and Ogliarola salentina samples showed a trend according to the cultivars. The two models built with the same number of component (1+1+0) gave a clear different predictive capability with Q^2^ = 0.838 and 0.509 for Dentamet®-treated (Figure 3a) and untreated (Figure 3b) Ogliarola salentina and Cellina di Nardò samples respectively. The higher predictive parameter value for treated samples revealed a stronger cultivar separation for treated with respect to untreated samples, demonstrating the treatment related metabolic response to *X. fastidiosa* infection. This result is in accord with our previous work [19], in which metabolic uniformity of aqueous extracts profile from *X. fastidiosa* infected Ogliarola salentina and Cellina di Nardò samples was observed. Once again the new findings demonstrate that effect of *X. fastidiosa* infection could be predominant over the cultivar metabolic profiles difference. It should be noted that, in the present study, two different sampling periods were considered, that are responsible of separation along the orthogonal component t[o] for both cultivars.

### 2.4. Supervised Discriminant Analyses on Ogliarola Salentina Metabolic Profiles

**Figure 4:**
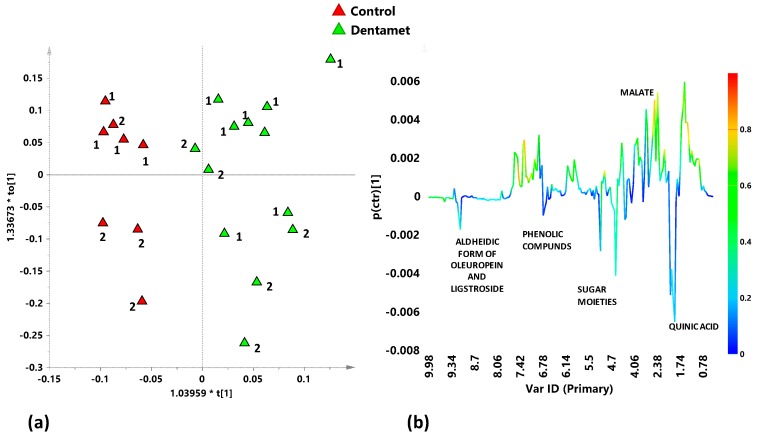
(**a**) OPLS-DA score plot for Ogliarola salentina treated with Dentamet® (green triangles) and untreated (Control) (red triangles) leaf extracts samples (1+3+0; R^2^X = 0.613; R^2^Y = 0.815; Q^2^ = 0.418); (**b**) *S*-line plot for the model, indicating molecular components responsible for the class separation. The corresponding predictive loadings are coloured according to the correlation scaled loading [*p*(*corr*)].

In order to investigate the response of each cultivar to Dentamet® treatment, metabolic profiles of Ogliarola salentina and Cellina di Nardò leaf samples were characterized separately. In particular, Ogliarola salentina treated with Dentamet® and untreated leaf samples, from Veglie and Copertino districts were studied with supervised discriminant analysis. The performed OPLS-DA gave a quite good model with Q^2^ = 0.418, revealing a separation according to the treatment application (Figure 4a). The molecules responsible of the class separation were indicate in the *S*-line plot for the model (Figure 4b). Ogliarola salentina leaf samples from infected leaves samples were characterized by higher relative content of quinic acid (signal at 1.94 ppm), α and β glucose (5.20 and 4.60 ppm), and aldehydic forms of oleuropein and ligstroside [range of δ = 9.0–9.5 ppm].

**Figure 5:**
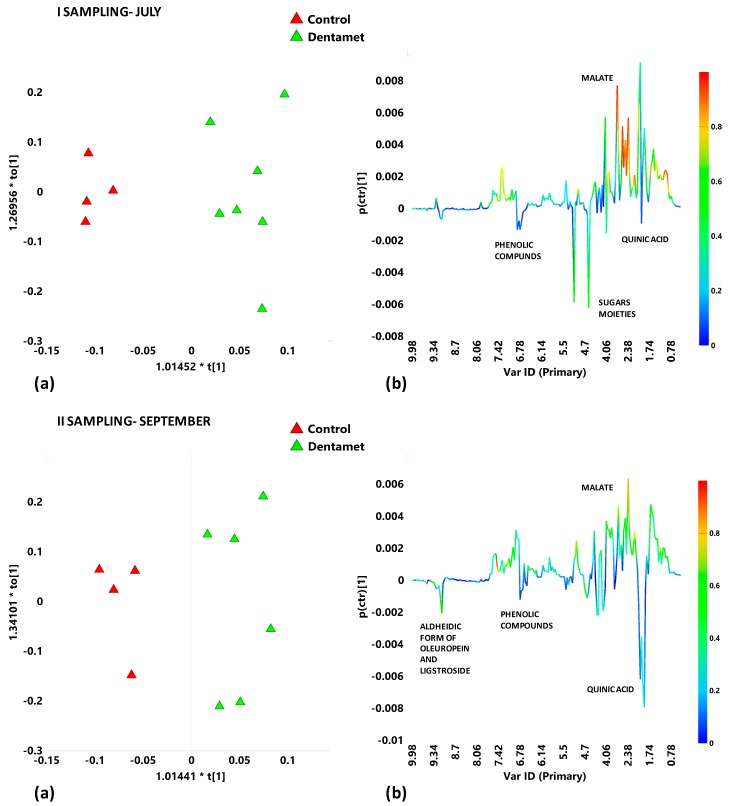
(**a**) OPLS-DA models score plots and (**b**) *S-line* plots for Dentamet® treated (green triangles) and untreated (Control) (red triangles) for Ogliarola salentina leaf extracts in the first (July) and second (September) samplings. Model parameters were: 1+3+0; R^2^X = 0.823; R^2^Y = 0.928; Q^2^ = 0.586; I sampling, July, and 1+3+0; R^2^X = 0.857; R^2^Y = 0.9; Q^2^ = 0.0414, II sampling, September. Molecular components responsible for the class separation could be observed in the *S*-line plots. The corresponding predictive loadings are coloured according to the correlation scaled loading [*p*(*corr*)].

Each sampling period was then analyzed with the aim to observe the effect of the Dentamet® on the two cultivars in the early stage of treatment. The discriminant supervised analysis performed on the Ogliarola salentina infected leaf samples collected in the first sampling (July) gave a quite good model (1+3+0; R^2^X = 0.823; R^2^Y = 0.928; Q^2^ = 0.586) in which a good separation between Dentamet®-treated and untreated samples could be observed (Figure 5a, I sampling). The *S*-line plot for the model revealed the difference into the metabolic profiles of the two considered classes of samples: quinic acid, sugars moieties (glucose in the two aqueous solution forms α and β), phenolic compounds (as phenylpropanoids derivatives) characterized the untreated samples (Figure 5b, I sampling).

In the following sampling period (II), the performed OPLS-DA gave a model (Figure 5a, II sampling) with lower predictive capability (1+3+0; R^2^X = 0.857; R^2^Y = 0.9; Q^2^ = 0.0414) with respect to the first sampling model. The molecular component observed to be responsible for the discrimination between groups were quinic acid, phenolic compounds and aldehydic forms of oleuropein and ligstroside for untreated samples (Figure 5b, II sampling).

Both the Dentamet®-treated leaf samples from Ogliarola salentina trees taken from Veglie and Copertino showed, in both sampling data, a higher content of malic acid (signals at 2.70 and 2.38 ppm) thus resulting this organic acid discriminative from the Ogliarola salentina trees when treated with this compound. It should be noted that, as reported in the experimental section, value of the central chemical shift for its specific 0.04 ppm is labelled in the bucket reduced spectra.

### 2.5. Supervised Discriminant Analyses on Cellina di Nardò Metabolic Profiles

**Figure 6:**
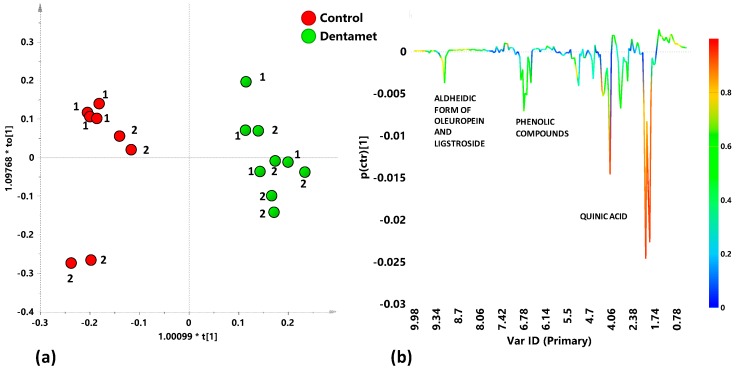
(**a**) OPLS-DA score plot for Cellina di Nardò treated with Dentamet® (green circles) and untreated (Control) (red circles) leaf extracts samples (1+1+0; R^2^X = 0.567; R^2^Y = 0.957; Q^2^ = 0.897); (**b**) *S-line* plot for the model, indicating molecular components responsible for the class separation. The corresponding predictive loadings are coloured according to the correlation scaled loading [*p*(*corr*)].

Supervised OPLS-DA analysis performed on Cellina di Nardò Dentamet® treated and untreated leaf samples, taken from Martina Franca and Nardò districts, gave a good model (1+1+0) with significant predictive capability Q^2^ = 0.897. A clear discrimination between treated and untreated class samples could be observed in the score plot (Figure 6a). The corresponding *S* line plot (Figure 6b) showed a significant higher relative content of quinic acid in untreated samples. Moreover, polyphenols molecules such as phenylpropanoids derivatives and aldehydic forms of oleuropein and ligstroside (signals at 6.78 and 9.14 ppm), present in higher levels, also characterized the untreated with respect to treated samples.

**Figure 7:**
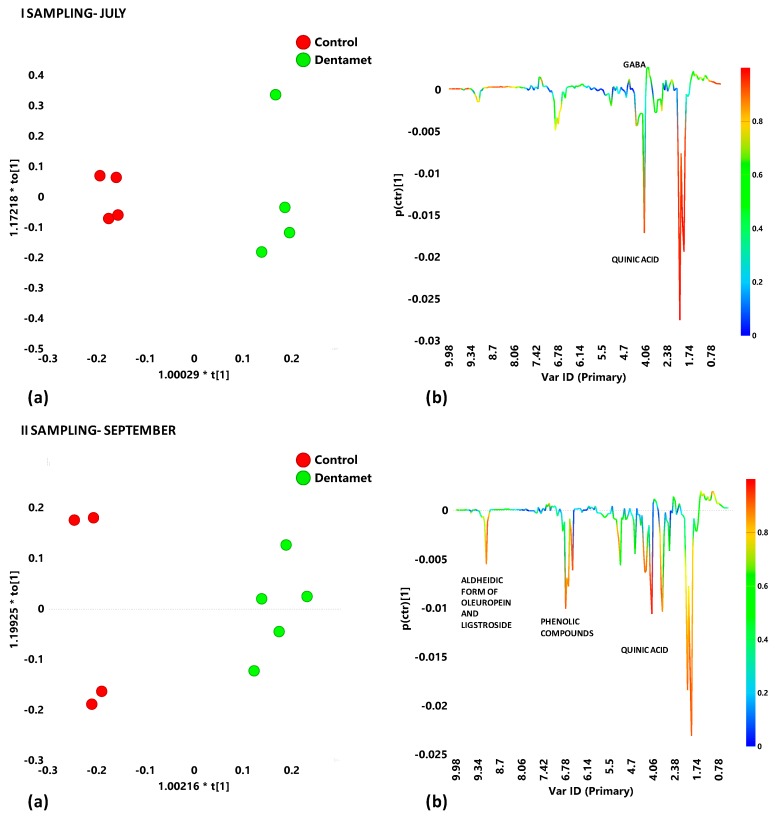
(**a**) OPLS-DA models score plots and (**b**) *S-line* plots for Dentamet® treated (green circles) and untreated (Control) (red circles) for Cellina di Nardò leaf extracts in the first (July) and second (September) samplings; Model parameters were (1+1+0; R^2^X = 0.742; R^2^Y = 0.989; Q^2^ = 0.945; I sampling, July) and (1+1+0; R^2^X = 0.697; R^2^Y = 0.974; Q^2^ = 0.9, II sampling, September). Molecular components responsible for the class separation could be observed in the *S*-line plots. The corresponding predictive loadings are coloured according to the correlation scaled loading [*p*(*corr*)].

The effect of Dentamet® treatment on Cellina di Nardò leaf samples was then analyzed by OPLS-DA analysis for each sampling period. For the first sampling period (July), the analysis gave a very good model (1+1+0; R^2^X = 0.742; R^2^Y = 0.989; Q^2^ = 0.945). Visual inspection of the t1/to[1] score plot of the model allowed to observe a marked separation between the two considered classes (Figure 7a, I sampling). Quinic acid was discriminating for all the untreated samples, whereas the occurrence of GABA characterized the Dentamet®-treated leaf samples (Figure 7b, I sampling). In the following sampling period (II), September, OPLS-DA analysis confirmed the separation between treated and untreated leaf samples with a good model (1+1+0; R^2^X = 0.697; R^2^Y = 0.974; Q^2^ = 0.9) (Figure 7a, II sampling). The *S-line* plot for the model revealed a general increase of metabolites such as quinic acid, phenolic compounds and aldehydic forms of oleuropein and ligstroside for untreated with respect to treated samples (Figure 7b, II sampling).

Interestingly, the Dentamet® -treated Cellina di Nardò leaf samples taken from Martina Franca and Nardò showed upon the OPLS-DA analysis, for both sampling periods, an attenuated metabolic response when compared with the untreated infected by *X. fastidiosa* subsp. *pauca*, samples. This also suggests a metabolic reprogramming of the tree metabolism upon the treatment. Indeed, the Dentamet® -treated Cellina di Nardò leaf samples also showed the occurrence of increased levels of GABA during the first sampling. On the other hand, in the treated leaf samples, the presence of increased levels of malate observed for Ogliarola salentina was never observed for the Cellina di Nardò.

## 3. Discussion

This study indicated a relevant variability of plant metabolites assessed by a ^1^H-NMR approach in relation to *X. fastidiosa* subsp. *pauca* infection and upon a crown treatment with a zinc-copper-citric acid biocomplex, namely Dentamet®. The two olive cultivars tested showed a similar response to the infection but a distinct response to the treatment. Both Ogliarola salentina and Cellina di Nardò untreated infected trees, irrespective of the site of sampling, showed a relative higher content of phenolic compounds and aldehydic forms of oleuropein and ligstroside when compared to the Dentamet®-treated samples in both samplings of July and September, thus confirming previous investigations [19]. As known, production and accumulation of aromatic secondary metabolites by various stresses is common in higher plants [31,32,33,34]. Plants synthesized a wide variety of secondary metabolites from primary metabolites (e.g., carbohydrates, lipids and amino acids) as a defense against herbivores, pathogens and environmental stresses [35,36]. Nevertheless, different level of pathogen attack together with several factors such as sampling period, cultivation area, cultivar seemed to be strictly correlated with polyphenols profiles [37].

Previously, Wallis and Chen [12] found in grapevine, grown in a climate-controlled greenhouse, artificially-infected with *X. f.* subsp. *fastidiosa*, a large-scale induction of phenolic compounds until two months from inoculation, which occurred in November, followed by a marked decrease of such metabolites. Subsequently, six months after inoculation, the infected plants showed marked sign of decline since the infection progression impeded the host to synthesize these secondary metabolites involved in the plant defence mechanisms. In our study, we did not exactly know when the infection process started, however, in the orchards where the occurrence of *X. fastidiosa* subsp. *pauca* was ascertained, we found an increase of phenolic compounds in leaves during both spring and summer. It should be said that olive is an evergreen species in which the mechanisms of *X. fastidiosa* induced secondary metabolites production could be different. Indeed such a production could initiate at each new season while the tree remains alive. It should be noted that in both Veglie and Martina Franca orchards the bacterium was officially recorded at least two years before this study. On the other hand, both Copertino and Nardò orchards already showed clear sign of infection (i.e., leaves and twig dieback) two years before the starting of this study. However, during the years following this study, the untreated trees showed a relevant increase of decline symptoms.

In the first sampling, Ogliarola salentina trees showed a higher relative content of sugar moieties (α and β glucose) with respect to the Cellina di Nardò in the untreated samples. Increase in glucose was already suggested as a result of a cascade of defence responses against disease and wounding involving shifts in carbohydrate metabolism with the aim to increase carbon uptake [25].

A marked enhancement in the carbon rich secondary metabolite quinic acid was observed in untreated Ogliarola salentina and Cellina di Nardò leaf samples in both sampling periods for all studied orchards. This results is in agreement with previous findings suggesting that the stress-associated metabolite quinic acid is abundant among metabolites induced after wounding in the genus *Quercus* [25]. The first direct ^1^H NMR observation of quinic acid in leaf samples extracts of naturally infected Ogliarola salentina and Cellina di Nardò is very interesting. This molecule has been recently found by HPLC MS and indicated as a possible marker for *X. fastidiosa* subsp. *pauca* infection in olive trees for the same cultivars [13]. Interestingly, the naturally infected Leccino cultivar, exhibiting more tolerance to *X. fastidiosa* subsp. *pauca* [13], shows, during the vegetative season, a higher content of quinic acid in comparison to Cellina di Nardò [14]. Moreover, greater levels of quinic acid were also observed in *X. fastidiosa*-infected *vs.* non-infected grapevines [12]. Quinic acid is, in fact, a precursor in lignification that is proposed as a general mechanism of plant resistance to biotic stress [14,38]. The present study therefore confirms the presence of a higher content of quinic acid in olive trees naturally infected by *X. fastidiosa* subsp. *pauca* and could suggest the use of this molecule as biomarker for the disease. Nevertheless concerning as also observed in the present study for Ogliarola salentina and Cellina di Nardò infected trees, the higher production of quinic acid does not prevent the infection to proceed over the years [39].

Ogliarola salentina trees treated with Dentamet® showed an higher content of malate with respect to untreated ones. Organic acids are, in general, synthesized and accumulated by plants because involved in osmotic regulation in drought stress situation [40,41]. Malic acid, in particular, is the most common organic acid in plant tissues in which it performs as a substrate for various cellular functions such as aminoacids biosynthesis and citric acid cycle [17]. Modifications in malate content was reported to be correlated with the plant response to stress condition. Malate is implicated in several metabolic pathways such as that involving the NADP-malic enzyme. It is therefore linked to both the defense-related deposition of lignin and the synthesis of reactive oxygen species produced for microbial pathogens inactivation [42]. In a previous NMR-based metabolomic analysis study, a decrease in malate content in the Huanglongbing (HLB) symptomatic leaves extracts was observed. The variation in malate content may indicate the higher consumption of this metabolite to support the normal functions of the citric acid cycle [17]. 

Leaves extract samples of Cellina di Nardò treated with Dentamet® showed with respect to untreated, a higher relative content of γ-aminobutyrate (GABA) in the July sampling. A rapid accumulation in plant tissues of high level of GABA was already observed as a response to environmental strain and considered as an adaptive stress mitigating factor [43,44]. Accumulation of GABA in symptomatic leaves of Pierce’s disease affected *Vitis vinifera* as a reaction for water, oxidative, and wounding stress in *Arabidopsis thaliana* was also recently found [34]. It was also suggested that GABA accumulation could provide a source of carbon skeletons for the TCA cycle in stress conditions inducing phenylpropanoids metabolism [43]. As known, phenylpropanoids metabolism provides many other metabolites, formed by phenylalanine [43] considered as indicators of plant stress responses [36,45]. These secondary metabolites are involved in plant defence mechanism such as cell thickening and lignin biosynthesis [34].

The present study, revealed that after few months from the crown treatment performed with a zinc-copper-citric acid biocomplex, a substantial change in the metabolic profiles of Ogliarola salentina and Cellina di Nardò leaves extracts samples was observed with respect to untreated. This change was observed in both of the samplings periods (July, September) and irrespective of the orchard location. The major observed differences relates to quinic acid and phenolic compounds exhibiting higher levels in untreated with respect to treated leaf samples. Higher malate and GABA were also observed in treated Ogliarola salentina and Cellina di Nardò leaf samples respectively. This metabolomic approach could buttress a previous study indicating Dentamet® as capable to significantly reduce the *X. fastidiosa* subsp. *pauca* cell density within Ogliarola salentina and Cellina di Nardò leaves and mitigate field symptoms [18]. In this study, the metabolomic analysis is in agreement with the disease trend observed in the four olive orchards. Indeed, the infection at the September survey, irrespective of the considered cultivar, resulted in a higher incidence for the untreated plots, thus confirming the effectiveness of Dentamet® in reducing the disease impact on the tree. However, the confirming of the mitigation of field symptoms coupled with the maintaining of a metabolic profile typical for trees hosting a pathogen cell number much lower than the untreated plants requires further investigation in the long-term period.

## 4. Materials and Methods 

### 4.1. Leaf Samples Collection

Samplings were carried out in four olive orchards planted with Ogliarola salentina or Cellina di Nardò and located in districts of the infected area of Lecce and Taranto provinces (Salento Peninsula, Apulia, southern Italy). Trees had an age ranging from 60 to 80 years. In all orchards, a plot of trees was sprayed on the canopy with Dentamet®, at a dose of 3.9 l per hectare, once per month, starting from early April 2017 to early September 2017, by using an atomizer [18]. At the beginning of the trial, the mean severity of symptoms (i.e., leaf and twig diebacks) scored in the plots treated with Dentamet® ranged from 10 to 20%. Control trees did not receive any treatment and showed, at the beginning of trial, the same severity as the plot including the Dentamet®-treated trees. A total of 40 leaf samples were collected in two sampling periods (early July and late September 2017) as follows: 12 Ogliarola Salentina leaf samples from 4 Dentamet®-treated and 2 untreated olive trees located at Veglie (Lecce province); 10 Ogliarola Salentina leaf samples from 3 Dentamet®-treated and 2 untreated olive trees located at Copertino (Lecce province); 10 Cellina di Nardò leaf samples from 3 Dentamet®-treated and 2 untreated olive trees at Martina Franca (Taranto province) and 8 Cellina di Nardò leaves samples from 2 Dentamet®-treated and 2 untreated olive trees at Nardò (Lecce province). Leaf samples, of 20 leaves each, were randomly taken from the crown paying attention at not taking withered leaves. The samples were put in plastic bags into a refrigerated box. In each farm, the occurrence of *X. fastidiosa* was molecularly assessed by following procedures previously described [19]. In the farms of Martina Franca and Veglie the occurrence of *X. fastidiosa* was also previously ascertained by the surveys carried out by the Phytosanitary Service of Apulia Region and subsequent analyses performed in the accredited laboratories of Apulia region. 

### 4.2. Sample Preparation for ^1^H NMR Analysis 

Samples were prepared according to the experimental procedure as reported in literature [46]. Briefly, olive leaf samples (each one containing 20 leaves) were plunged into liquid N_2_ and ground to a fine powder with a stainless steel blender. Ground leaves were transferred into a plastic tube and placed in a freeze dryer for 48 h. Lyophilized plant material (100 mg) was weighted into an autoclaved 2 mL Eppendorf tube. Thereafter, 0.75 mL of CD_3_OD and 0.75 mL of KH_2_PO_4_ buffer in D_2_0 (pH 5.9) containing 0.05% *w*/*v* TSP-*d*4 (sodium salt of trimethylsilylpropionic acid) were added to each sample. The content of the eppendorf tubes was mixed thoroughly with a vortex mixer at room temperature for 1 minute and then sonicated for 10 minutes at room temperature. Samples were spun down in a microcentrifuge at 17,000× *g* for 20 min; then, 700 µL of the supernatant were filled into a 5 mm NMR tube.

### 4.3. ^1^H-NMR Fingerprinting and Metabolite Identification

All measurements were performed on a Bruker Avance III 600 Ascend NMR spectrometer (Bruker, Ettlingen, Germany), operating at 600.13 MHz for ^1^H observation, equipped with a TCI cryoprobe incorporating a z axis gradient coil and automatic tuning-matching (ATM). Experiments were acquired at 300 K in automation mode after loading individual samples by a Bruker Automatic Sample Changer, interfaced with the software IconNMR (Bruker). For each sample a 1D sequence with pre-saturation and composite pulse for selection (zgcppr Bruker standard pulse sequence) was acquired, with 16 transients, 16 dummy scans, 5 s relaxation delay, size of fid of 64K data points, a spectral width of 12019.230 Hz (20.0276 ppm) and an acquisition time of 2.73 s. The resulting FIDs were multiplied by an exponential weighting function corresponding to a line broadening of 0.3 Hz before Fourier transformation, automated phasing and baseline correction. Metabolite identification were based on ^1^H and ^13^C assignment by 1D and 2D omo and eteronuclear experiments and by comparison with literature data [19,26,47,48,49]. NMR data processing was performed by using TopSpin 3.5 (Bruker). All spectra were referenced to the TSP signal (δ = 0.00 ppm).

### 4.4. ^1^H NMR Data Processing and Multivariate Statistical Analyses

^1^H NMR spectra were converted to a suitable form for multivariate analysis by segmentation into small rectangular bins (buckets) and integration by Amix 3.9.15 (Analysis of Mixture, Bruker BioSpin GmbH, Rheinstetten, Germany) software. Bucketing was performed within 10.00–0.5 ppm region, excluding the residual non-deuterated water (4.9–4.7 ppm) and methanol (3.40–3.30 ppm) signals. In order to account for small pH or concentration signals shift, the width of each bucket was fixed to 0.04 ppm. The total sum normalization was then performed in order to minimize small differences due to metabolites concentration and/or experimental conditions among samples [50,51,52]. The Pareto scaling method, which is performed by dividing the mean-centered data by the square root of the standard deviation, was then applied to the bucket reduced NMR spectra (variables). The data table arisen from all aligned buckets row reduced spectra was used for further multivariate data analysis. Each bucket row depicts the entire NMR spectrum, with all the molecules present in the sample. Moreover, each bucket in a buckets row reduced spectrum is labeled with the value of the central chemical shift for its specific 0.04 ppm width. The buckets are the variables used as descriptors for each sample in chemometric analyses. Software Simca-P version 14 (Sartorius Stedim Biotech, Umeå, Sweden) was used to accomplish multivariate statistical analysis. In particular, PCA (Principal Component Analysis), PLS-DA (data not shown) and OPLS-DA (Partial Least Squares and Orthogonal Partial Least Squares Discriminant Analyses, respectively) were perfomed [53,54,55]. Principal Component Analysis is at the basis of the multivariate statistical analysis [53] and usually applied to the data to extract and show the systematic variation in a data matrix X formed by rows (the considered observations), in our case the leaves extract samples and columns (the variables which describe the samples), in our case the buckets from each NMR spectrum [56]. Moreover, by PCA, groups of observations, trends and outliers that could be excluded from further analysis may be recognized. In this work, the PLS-DA analysis was also applied with the aim to justify the number of components used in OPLS-DA model [57]. The OPLS-DA analysis is a modification of the usual PLS-DA method which filters out variation not directly related to the discriminating response and brings out models of clearer interpretation, as reported in several studies [58,59]. The further refinement provide by the OPLS-DA in MVA lies in the capability to divide the portion of the variance helpful for predictive purposes from the not predictive variance (which is made orthogonal) [60]. The robustness and predictive ability of the statistical models was estimated by a seven-fold cross-validation procedure [61,62,63]. The analysis of R^2^ and Q^2^ parameters can easily define the minimal number of required components. These parameters show completely different behavior with the model complexity increase. The R^2^X, R^2^Y and Q^2^, describing the total variation in X, the variation in the response variable Y and the predictive ability of the models, respectively, were calculated [64]. The results were represented by the optimal (PCA) or more significant (OPLS-DA) bidimensional scores plots and relative loadings plots, by which differences among groups could be recognized [57,65,66].

### 4.5. Chemicals

All chemical reagents for analysis were of analytical grade. Deuterium oxide (99.9 atom %D) containing 0.05% wt 3-(trimethylsilyl)propionic-2,2,3,3 *d4* acid sodium salt (TSP), Potassium phosphate monobasic were purchased from Armar Chemicals (Döttingen, Switzerland). Methanol-*d4* (99.9 atom %D), potassium phosphate monobasic were purchased from CARLO ERBA Reagents (Milano, Italia).

## 5. Conclusions

In this study, a ^1^H-NMR-based metabolomic approach was used to provide a snapshot of the plant–pathogen interaction that modifies the plant metabolic pathway, and to identify potential biomarkers of *X. fastidiosa* subsp. *pauca* infection to olive trees at its early stages. The metabolic response to the infection was analyzed by considering untreated and trees treated with zinc-copper-citric acid. The effect of the treatment could be clearly studied by analyzing the metabolic profiles and following, in the overall metabolites change response, a well known disease marker such as quinic acid. Notwithstanding, further investigations are necessary to confirm these findings also in the long-term period. These results could be useful for the development of strategies for infection containment and plant health promotion. The suitability of ^1^H-NMR metabolic profiling approaches to monitor and characterize the metabolome shift in response to stress such as pathogen infection was confirmed.

## Figures and Tables

**Table 1:** Mean severity index observed in Ogliarola salentina and Cellina di Nardò olive trees and assessed by visually scoring, in each plot, the percentage of wilted leaves and twigs per tree.

Location		Copertino (LE)		Veglie (LE)		Nardò (LE)		Martina Franca (TA)	
Cultivar		Ogliarola salentina		Ogliarola salentina		Cellina di Nardò		Cellina di Nardò	
Initial mean severity index (%)		10		20		20		10	
Sampling periods (2017)		July	September	July	September	July	September	July	September
Mean severity index (%)	Untreated (Control)	15	20	25	25	25	30	10	15
	Treated (Dentamet®)	10	10	20	20	20	20	10	10

## References

[1] Purcell A. (2013). Paradigms: Examples from the bacterium *Xylella fastidiosa*. Annu. Rev. Phytopathol..

[2] Schaad N.W., Postnikova E., Lacy G., Fatmi M.B., Chang C.-J. (2004). *Xylella fastidiosa* subspecies: *X. fastidiosa* subsp *piercei*, subsp. nov., *X. fastidiosa* subsp. multiplex subsp. nov., and X. fastidiosa subsp. pauca subsp. nov. Syst. Appl. Microbiol..

[3] Denancé N., Briand M., Gaborieau R., Gaillard S., Jacques M.-A. (2019). Identification of genetic relationships and subspecies signatures in *Xylella fastidiosa*. BMC Genom..

[4] Marcelletti S., Scortichini M. (2016). Genome-wide comparison and taxonomic relatedness of multiple *Xylella fastidiosa* strains reveal the occurrence of three subspecies and a new Xylella species. Arch. Microbiol..

[5] Martelli G., Boscia D., Porcelli F., Saponari M. (2016). The olive quick decline syndrome in south-east Italy: A threatening phytosanitary emergency. Eur. J. Plant Pathol..

[6] Cobine P.A., Cruz L.F., Navarrete F., Duncan D., Tygart M., De La Fuente L. (2013). *Xylella fastidiosa* differentially accumulates mineral elements in biofilm and planktonic cells. PLoS ONE.

[7] Navarrete F., De La Fuente L. (2014). Response of *Xylella fastidiosa* to zinc: Decreased culturability, increased exopolysaccharide production, and formation of resilient biofilms under flow conditions. Appl. Environ. Microbiol..

[8] Oliver J., Cobine P., De La Fuente L. (2015). *Xylella fastidiosa* isolates from both subsp. multiplex and fastidiosa cause disease on southern highbush blueberry (*Vaccinium* sp.) under greenhouse conditions. Phytopathology.

[9] Scortichini M., Migoni D., Angilè F., Del Coco L., Girelli C.R., Zampella L., Mastrobuoni F., Fanizzi F.P. (2019). *Xylella fastidiosa* subsp. *pauca* on olive in Salento (Southern Italy): Infected trees have low in planta micronutrient content. Phytopathol. Mediterr..

[10] Wallis C.M., Chen J. (2012). Grapevine phenolic compounds in xylem sap and tissues are significantly altered during infection by *Xylella fastidiosa*. Phytopathology.

[11] Wallis C.M., Wallingford A.K., Chen J. (2013). Effects of cultivar, phenology, and *Xylella fastidiosa* infection on grapevine xylem sap and tissue phenolic content. Physiol. Mol. Plant Pathol..

[12] Wallis C.M., Wallingford A.K., Chen J. (2013). Grapevine rootstock effects on scion sap phenolic levels, resistance to *Xylella fastidiosa* infection, and progression of Pierce’s disease. Front. Plant Sci..

[13] Luvisi A., Aprile A., Sabella E., Vergine M., Nicoli F., Nutricati E., Miceli A., Negro C., De Bellis L. (2017). *Xylella fastidiosa* subsp. *pauca* (CoDiRO strain) infection in four olive (*Olea europaea* L.) cultivars: Profile of phenolic compounds in leaves and progression of leaf scorch symptoms. Phytopathol. Mediterr..

[14] Sabella E., Luvisi A., Aprile A., Negro C., Vergine M., Nicolì F., Miceli A., De Bellis L. (2018). *Xylella fastidiosa* induces differential expression of lignification related-genes and lignin accumulation in tolerant olive trees cv. Leccino. J. Plant Physiol..

[15] Deborde C., Moing A., Roch L., Jacob D., Rolin D., Giraudeau P. (2017). Plant metabolism as studied by NMR spectroscopy. Prog. Nucl. Magn. Reson. Spectrosc..

[16] Leiss K.A., Choi Y.H., Verpoorte R., Klinkhamer P.G. (2011). An overview of NMR-based metabolomics to identify secondary plant compounds involved in host plant resistance. Phytochem. Rev..

[17] dos Santos Freitas D., Carlos E.F., Gil M.r.C.S.d.S., Vieira L.G.E., Alcantara G.B. (2015). NMR-Based Metabolomic analysis of Huanglongbing-asymptomatic and-symptomatic citrus trees. J. Agric. Food Chem..

[18] Scortichini M., Chen J., De Caroli M., Dalessandro G., Pucci N., Modesti V., L’Aurora A., Petriccione M., Zampella L., Mastrobuoni F. (2018). A zinc, copper and citric acid biocomplex shows promise for control of *Xylella fastidiosa* subsp. pauca in olive trees in Apulia region (southern Italy). Phytopathol. Mediterr..

[19] Girelli C.R., Del Coco L., Scortichini M., Petriccione M., Zampella L., Mastrobuoni F., Cesari G., Bertaccini A., D’amico G., Contaldo N. (2017). *Xylella fastidiosa* and olive quick decline syndrome (CoDiRO) in Salento (southern Italy): A chemometric ^1^ H NMR-based preliminary study on Ogliarola salentina and Cellina di Nardò cultivars. Chem. Biol. Technol. Agric..

[20] Jacobs J.M., Babujee L., Meng F., Milling A., Allen C. (2012). The in planta transcriptome of *Ralstonia solanacearum*: Conserved physiological and virulence strategies during bacterial wilt of tomato. MBio.

[21] Pieretti I., Royer M., Barbe V., Carrere S., Koebnik R., Couloux A., Darrasse A., Gouzy J., Jacques M.-A., Lauber E. (2012). Genomic insights into strategies used by *Xanthomonas albilineans* with its reduced artillery to spread within sugarcane xylem vessels. BMC Genom..

[22] Fatima U., Senthil-Kumar M. (2015). Plant and pathogen nutrient acquisition strategies. Front. Plant Sci..

[23] Rahmanian N., Jafari S.M., Wani T.A. (2015). Bioactive profile, dehydration, extraction and application of the bioactive components of olive leaves. Trends Food Sci. Technol..

[24] Killiny N. (2017). Metabolite signature of the phloem sap of fourteen citrus varieties with different degrees of tolerance to *Candidatus* Liberibacter asiaticus. Physiol. Mol. Plant Pathol..

[25] Sardans J., Gargallo-Garriga A., Pérez-Trujillo M., Parella T., Seco R., Filella I., Penuelas J. (2014). Metabolic responses of *Quercus ilex* seedlings to wounding analysed with nuclear magnetic resonance profiling. Plant Biol..

[26] Goulas V., Exarchou V., Troganis A.N., Psomiadou E., Fotsis T., Briasoulis E., Gerothanassis I.P. (2009). Phytochemicals in olive-leaf extracts and their antiproliferative activity against cancer and endothelial cells. Mol. Nutr. Food Res..

[27] Lee O.-H., Lee B.-Y., Lee J., Lee H.-B., Son J.-Y., Park C.-S., Shetty K., Kim Y.-C. (2009). Assessment of phenolics-enriched extract and fractions of olive leaves and their antioxidant activities. Bioresour. Technol..

[28] Del Coco L., De Pascali S., Fanizzi F.P. (2014). ^1^H NMR spectroscopy and multivariate analysis of monovarietal EVOOs as a tool for modulating Coratina-based blends. Foods.

[29] Krishnan P., Kruger N., Ratcliffe R. (2004). Metabolite fingerprinting and profiling in plants using NMR. J. Exp. Bot..

[30] Rivas-Ubach A., Pérez-Trujillo M., Sardans J., Gargallo-Garriga A., Parella T., Penuelas J. (2013). Ecometabolomics: Optimized NMR-based method. Methods Ecol. Evol..

[31] Karioti A., Chatzopoulou A., Bilia A.R., Liakopoulos G., Stavrianakou S., Skaltsa H. (2006). Novel secoiridoid glucosides in *Olea europaea* leaves suffering from boron deficiency. Biosci. Biotechnol. Biochem..

[32] Romero M.P., Tovar M.J., Ramo T., Motilva M.J. (2003). Effect of crop season on the composition of virgin olive oil with protected designation of origin “Les Garrigues”. J. Am. Oil Chem. Soc..

[33] Sofo A., Dichio B., Xiloyannis C., Masia A. (2005). Antioxidant defences in olive trees during drought stress: Changes in activity of some antioxidant enzymes. Funct. Plant Biol..

[34] Zaini P.A., Nascimento R., Gouran H., Cantu D., Chakraborty S., Phu M., Goulart L.R., Dandekar A.M. (2018). Molecular Profiling of Pierce’s Disease Outlines the Response Circuitry of *Vitis vinifera* to *Xylella fastidiosa* Infection. Front. Plant Sci..

[35] Akula R., Ravishankar G.A. (2011). Influence of abiotic stress signals on secondary metabolites in plants. Plant Signal. Behav..

[36] Guodong R., Xiaoxia L., Weiwei Z., Wenjun W., Jianguo Z. (2017). Metabolomics reveals variation and correlation among different tissues of olive (*Olea europaea* L.). Biol. Open.

[37] Abaza L., Taamalli A., Nsir H., Zarrouk M. (2015). Olive tree (*Olea europeae* L.) leaves: Importance and advances in the analysis of phenolic compounds. Antioxidants.

[38] Barros J., Serk H., Granlund I., Pesquet E. (2015). The cell biology of lignification in higher plants. Ann. Bot..

[39] Giampetruzzi A., Morelli M., Saponari M., Loconsole G., Chiumenti M., Boscia D., Savino V.N., Martelli G.P., Saldarelli P. (2016). Transcriptome profiling of two olive cultivars in response to infection by the CoDiRO strain of Xylella fastidiosa subsp. pauca. BMC Genom..

[40] Bianco R., Avellone G. (2014). Diurnal regulation of leaf water status in high-and low-mannitol olive cultivars. Plants.

[41] Dichio B., Xiloyannis C., Sofo A., Montanaro G. (2006). Osmotic regulation in leaves and roots of olive trees during a water deficit and rewatering. Tree Physiol..

[42] Casati P., Drincovich M.F., Edwards G.E., Andreo C.S. (1999). Malate metabolism by NADP-malic enzyme in plant defense. Photosynth. Res..

[43] Kinnersley A.M., Turano F.J. (2000). Gamma aminobutyric acid (GABA) and plant responses to stress. Crit. Rev. Plant Sci..

[44] Bown A.W., Shelp B.J. (2016). Plant GABA: Not just a metabolite. Trends Plant Sci..

[45] Dixon R.A., Paiva N.L. (1995). Stress-induced phenylpropanoid metabolism. Plant Cell.

[46] Kim H.K., Choi Y.H., Verpoorte R. (2010). NMR-based metabolomic analysis of plants. Nat. Protoc..

[47] Christophoridou S., Dais P., Tseng L.-H., Spraul M. (2005). Separation and identification of phenolic compounds in olive oil by coupling high-performance liquid chromatography with postcolumn solid-phase extraction to nuclear magnetic resonance spectroscopy (LC-SPE-NMR). J. Agric. Food Chem..

[48] Christophoridou S., Dais P. (2009). Detection and quantification of phenolic compounds in olive oil by high resolution ^1^H nuclear magnetic resonance spectroscopy. Anal. Chim. Acta.

[49] Owen R.W., Mier W., Giacosa A., Hull W.E., Spiegelhalder B., Bartsch H. (2000). Identification of lignans as major components in the phenolic fraction of olive oil. Clin. Chem..

[50] Sundekilde U., Larsen L., Bertram H. (2013). NMR-based milk metabolomics. Metabolites.

[51] Gallo V., Mastrorilli P., Cafagna I., Nitti G.I., Latronico M., Longobardi F., Minoja A.P., Napoli C., Romito V.A., Schäfer H. (2014). Effects of agronomical practices on chemical composition of table grapes evaluated by NMR spectroscopy. J. Food Compos. Anal..

[52] van den Berg R.A., Hoefsloot H.C., Westerhuis J.A., Smilde A.K., van der Werf M.J. (2006). Centering, scaling, and transformations: Improving the biological information content of metabolomics data. BMC Genom..

[53] Jackson J.E. (2005). A User’s Guide to Principal Components.

[54] Eriksson L., Byrne T., Johansson E., Trygg J., Vikström C. (2013). Multi-and Megavariate DATA ANALYSIS BASIC principles and Applications.

[55] Lindon J.C., Nicholson J.K., Holmes E. (2011). The Handbook of Metabonomics and Metabolomics.

[56] Girelli C.R., Del Coco L., Fanizzi F.P. (2016). ^1^H NMR spectroscopy and multivariate analysis as possible tool to assess cultivars, from specific geographical areas, in evoos. Eur. J. Lipid Sci. Technol..

[57] Girelli C.R., De Pascali S.A., Del Coco L., Fanizzi F.P. (2016). Metabolic profile comparison of fruit juice from certified sweet cherry trees (*Prunus avium* L.) of Ferrovia and Giorgia cultivars: A preliminary study. Food Res. Int..

[58] Consonni R., Cagliani L., Benevelli F., Spraul M., Humpfer E., Stocchero M. (2008). NMR and chemometric methods: A powerful combination for characterization of balsamic and traditional balsamic vinegar of Modena. Anal. Chim. Acta.

[59] Girelli C.R., Del Coco L., Zelasco S., Salimonti A., Conforti F.L., Biagianti A., Barbini D., Fanizzi F.P. (2018). Traceability of “Tuscan PGI” Extra Virgin Olive Oils by ^1^H NMR Metabolic Profiles Collection and Analysis. Metabolites.

[60] Boccard J., Rutledge D.N. (2013). A consensus orthogonal partial least squares discriminant analysis (OPLS-DA) strategy for multiblock Omics data fusion. Anal. Chim. Acta.

[61] Holmes E., Loo R.L., Stamler J., Bictash M., Yap I.K., Chan Q., Ebbels T., De Iorio M., Brown I.J., Veselkov K.A. (2008). Human metabolic phenotype diversity and its association with diet and blood pressure. Nature.

[62] Trygg J., Wold S. (2002). Orthogonal projections to latent structures (O-PLS). J. Chemom. A J. Chemom. Soc..

[63] Triba M.N., Le Moyec L., Amathieu R., Goossens C., Bouchemal N., Nahon P., Rutledge D.N., Savarin P. (2015). PLS/OPLS models in metabolomics: The impact of permutation of dataset rows on the K-fold cross-validation quality parameters. Mol. Biosyst..

[64] Wheelock Å.M., Wheelock C.E. (2013). Trials and tribulations of ‘omics data analysis: Assessing quality of SIMCA-based multivariate models using examples from pulmonary medicine. Mol. Biosyst..

[65] Sun L., Zhang H., Wu L., Shu S., Xia C., Xu C., Zheng J. (2014). ^1^H-Nuclear magnetic resonance-based plasma metabolic profiling of dairy cows with clinical and subclinical ketosis. J. Dairy Sci..

[66] Girelli C.R., Accogli R., Del Coco L., Angilè F., De Bellis L., Fanizzi F.P. (2018). ^1^H-NMR-based metabolomic profiles of different sweet melon (*Cucumis melo* L.) Salento varieties: Analysis and comparison. Food Res. Int..

